# Lack of Evidence for Schmallenberg Virus Infection in Highly Exposed Persons, Germany, 2012

**DOI:** 10.3201/eid1808.120533

**Published:** 2012-08

**Authors:** Tanja Ducomble, Hendrik Wilking, Klaus Stark, Anja Takla, Mona Askar, Lars Schaade, Andreas Nitsche, Andreas Kurth

**Affiliations:** Robert Koch Institute, Berlin, Germany (T. Ducomble, H. Wilking, K. Stark, A. Takla, M. Askar, L. Schaade, A. Nitsche, A. Kurth);; European Program for Intervention Epidemiology Training, Stockholm, Sweden (T. Ducomble);; and Postgraduate Training for Applied Epidemiology–German Field Epidemiology Training Program, Berlin (A. Takla, M. Askar)

**Keywords:** Orthobunyavirus, zoonoses, seroepidemiologic studies, Schmallenberg virus, viruses, vector-borne infections, Germany

## Abstract

Schmallenberg virus, a novel orthobunyavirus, is spreading among ruminants, especially sheep, throughout Europe. To determine the risk for human infection, we conducted a survey among shepherds to assess possible exposure and symptoms. We also performed serologic and molecular assays. No evidence of transmission to humans was detected.

In November 2011, a new virus of the genus *Orthobunyavirus* was isolated from diseased cattle in Germany and was provisionally called Schmallenberg virus (SBV) (*1*). It has caused disease in ruminants, i.e., sheep, cattle, and goats. Acute clinical signs such as fever and diarrhea; severe congenital malformation, such as arthrogryposis and hydroencephaly; and a high proportion of stillbirths have been reported among infected animals (*2*). Transplacental transmission leads to fetal infection. The virus is vector borne and has been isolated from biting midges (*Culicoides* spp.) (*3**–**5*). Genomic analyses showed a close phylogenetic relationship to epizootic viruses of the Simbu serogroup, for which zoonotic transmission has not been shown (*1*). However, SBV also bears new genetic and animal-related clinical and epidemiologic properties. Iquitos and Oropouche viruses of this serogroup are also transmitted by culicoids and cause outbreaks in humans (*6*). La Crosse virus and California encephalitis virus can cause disease in humans and belong to the genus *Orthobunyavirus*. A few vector-borne zoonoses from the same family *Bunyaviridae*, i.e., Rift Valley fever virus and Crimean-Congo hemorrhagic fever virus, also are highly transmissible to humans through handling of infectious animal tissue. However, this mode of transmission has not been described for orthobunyaviruses. Shortly after its recognition, SBV and associated disease were reported from an increasing number of European countries, and further spread is conceivable. The virus currently is isolated mainly from sheep farms (*7**,**8*). In Germany, North Rhine-Westphalia is the area most affected. Viral loads are high in infected animals and their birth products (*2*). Thus, shepherds can be considered as strongly exposed, especially during animal obstetric events.

Because SBV emerged recently, transmission from animals to human cannot be completely excluded. Knowing whether SBV poses a risk to humans is vital. Therefore, we conducted a seroprevalence study among exposed shepherds in the area in Germany most affected (North Rhine-Westphalia) to determine whether zoonotic or vector-borne infections occur in humans.

## The Study

At an SBV information meeting, 60 shepherds >18 years of age were recruited for this study. After obtaining written informed consent, we administered a standardized questionnaire. We collected information about age, sex, SBV infection in their livestock, exposure to sick lambs, frequency of insect bites, personal health, and categories of signs of disease after exposure. In addition, a serum sample was taken from each participant.

We developed an indirect fluorescent antibody test (IFAT) for primary testing of human serum. For this test, antihuman fluorescein isothiocyanate–conjugated secondary antibodies against SBV-specific IgM or IgG (antibovine for positive control) were used. For the IFAT, all heat-inactivated serum specimens were tested in dilutions of 1:20 and 1:80 on glass slides with noninfected and SBV-infected Vero cells. An SBV antibody–positive serum sample from an experimentally infected cow was used as a positive control. To check for background signals and possible cross-reactivity, we tested 80 serum samples from healthy blood donors; none were positive. A serum neutralization test (SNT) was developed for confirmation of indeterminate and positive results. Serial dilutions of the test serum (lowest dilution 1:5) were incubated for 1 h at 37°C with an equal volume of cell culture supernatant containing 100 infectious doses of SBV and subsequently mixed with Vero cells. To detect SBV-specific RNA, we performed a 1-step real-time reverse transcription quantitative PCR (RT-qPCR) on serum, as described (*1*). The ethics committee of the University Medicine Charité Berlin approved our study.

All 60 participants (75% male; median age 48 years [interquartile range (IQR) 41–56 years]) reported sheep husbandry in the SBV-epizootic area (Table 1). Altogether, 48 (80%) participants had contact with lambs that had characteristic malformations or with the respective birth products (median 10 [IQR 4–20] sick lambs). In livestock from 36 (60%) participants, SBV was laboratory confirmed. Characteristic signs among adult animals had first been noted in September 2011. Median time from first signs in animals to blood withdrawal was 45 days (IQR 39–66 days). A total of 55 (98%) of 56 participants self-reported insect bites during late summer to autumn; among these, 22 (39%) indicated frequent insect bites. Nine (15%) shepherds reported having had signs and symptoms since the disease had appeared in the study area or after handling diseased animals (Table 1): myalgia and arthralgia (7 shepherds), headache (4), fever (4), skin rash (2), and respiratory problems (2). No shepherds reported hospitalization. Of the 36 shepherds whose livestock had laboratory-confirmed SBV infection, 5 (14%) reported signs and symptoms: myalgia and arthralgia (4 shepherds), headache (2), fever (2), skin rash (2), and respiratory problems (2).

**Table 1:** Self-reported exposure and symptoms of persons exposed to novel SBV, Germany, 2012*

Exposure category†	No. shepherds exposed/total no. (%)	No. shepherds with symptoms/total no. exposed (%)‡
Sheep husbandry in SBV-epizootic area	60/60 (100)	9/60 (15)
Laboratory-confirmed SBV infection in livestock	36/60 (60)	5/36 (14)
Contact with birth products or with lambs that had characteristic signs of SBV disease	48/60 (80)	8/48 (17)
Contact with adult sheep that had characteristic signs of SBV disease	28/51 (55)	5/28 (18)
Frequent insect bites in SBV-epizootic area§	22/56 (39)	5/22 (23)

*SBV, Schmallenberg virus. †Multiple responses possible. ‡Self-reported signs and symptoms of fever, headache, skin rash, myalgia/arthralgia, respiratory problems, or photophobia since SBV infection appeared in the study area or after handling diseased animals and resulting from unknown cause in each exposure category. §Self-reported as ‘”very often” or “often.”

No SBV-specific antibodies were detected in any serum specimens (Table 2). Eight specimens showed indeterminate fluorescent signals in the IFAT at a 1:20 serum dilution for IgG (n = 1) or IgM (n = 7) but were not reactive at 1:80 (Figure). These 8 samples were retested by SNT (serum dilution 1:10) and showed no virus inhibition at any serum dilution during 7 days of incubation. Two (25%) of these 8 shepherds reported symptoms. For the bovine control serum, the titer of the SNT was 320. RT-qPCR was negative in all serum samples.

**Table 2:** Results of diagnostic tests for SBV in serum samples from exposed shepherds, Germany, 2012*

Test system and dilution	Test results, no. (%)	
	Positive/total	Indeterminate/total
IFAT IgG 80	0/60 (0)	0/60 (0)
IFAT IgM 80	0/60 (0)	0/60 (0)
IFAT IgG 20	0/60 (0)	1/60 (2)
IFAT IgM 20	0/60 (0)	7/60 (11)
SNT titer 10†	0/8 (0)	0/8 (0)
RT-qPCR	0/60 (0)	0/60 (0)

*SBV, Schmallenberg virus; IFAT, indirect fluorescent antibody test; SNT, serum neutralization test; RT-qPCR, quantitative reverse transcription PCR. †Performed in only 8 serum samples with IFAT indeterminate results; 2/8 (25%) reported symptoms as outlined in Table 1.

**Figure:**
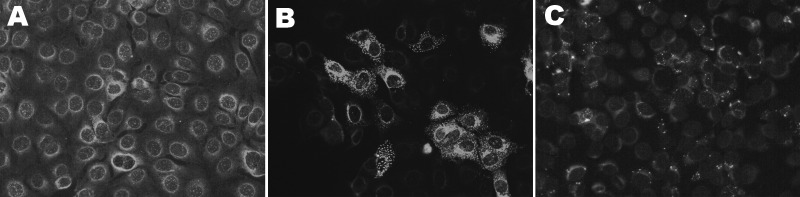
Fluorescent light microscopy images of serum samples tested for antibodies to Schmallenberg virus by indirect fluorescent antibody test on infected Vero cells mixed with noninfected Vero cells. A) Nonreactive negative serum; B) positive serum reactive with infected cells only (green); C) indeterminate serum with faint nonspecific reactivity. Colors have been enhanced to show detail.

## Conclusions

We investigated the risk for human infection after possible high exposure to an emerging vector-borne epizootic disease through contact with infected animals and tissues or through insect bites. No evidence of SBV infection among the shepherds was found by molecular and serologic tests, even though most of the shepherds had received substantial exposure through repeated direct contact with sheep with laboratory-confirmed SBV-infection and with birth products known to contain high virus loads in the SBV-epizootic area. Reported symptoms were compatible with illnesses commonly experienced during the winter (i.e., influenza-like illness caused by human respiratory viruses) without considerable differences between the exposure categories. The likelihood of virus detection by RT-qPCR is certainly limited because SBV viremia in livestock lasts only a few days (*4**,**8*). Viremia could be of short duration in humans as well. However, after the end of the viremic phase, detection of specific antibodies can be expected. The period between exposure and sampling was sufficiently long to for antibodies to have developed after infection. Furthermore, a large proportion of the participants indicated having been frequently bitten by insects in the epizootic area. Midge bites are difficult to recall, and therefore this exposure could not be assessed precisely. Recollection of insect bites might not be equivalent to exposure to the vector species. Although SBV has been isolated from certain midge species, entomologic knowledge about the ability of different midge species to transmit SBV, i.e., vector competence and host feeding behavior, is still scarce. Nevertheless, on the basis of results from our study and the phylogenetic relationship of SBV, we conclude that the novel virus is unlikely to pose a threat to humans by transmission from infected livestock or from midges.
